# Evaluating the Association Between Obesity and Development of Trigger Finger and Carpal Tunnel Syndrome

**DOI:** 10.1016/j.jhsg.2025.100760

**Published:** 2025-05-31

**Authors:** Jason Sidrak, Andy Lalka, Cailin Delaney, Frank Scott

**Affiliations:** ∗ University of Colorado Anschutz School of Medicine, Aurora CO; † Children’s Hospital Colorado, University of Colorado Anschutz Medical Center, Aurora CO; ‡ Department of Orthopedics, University of Colorado Anschutz Medical Center, Aurora CO

**Keywords:** Carpal tunnel syndrome, Compression injury of nerve, Hand orthopedic surgery, Obesity

## Abstract

**Purpose:**

Obesity is known to cause a low-grade inflammatory environment that can influence the development of musculoskeletal disorders. We hypothesized that patients with high body mass index (BMI), defined as ≥30, would develop carpal tunnel syndrome (CTS) or stenosing tenosynovitis (TF) earlier than patients with a normal BMI.

**Methods:**

A retrospective chart review was performed for adults diagnosed with CTS or TF between January 2016 and December 2020 at a tertiary center. The primary outcome was age at diagnosis of CTS or TF. A high BMI was considered ≥ 30, whereas a normal BMI was 18.5–24.9. A stepwise multivariate linear regression model compared the association of BMI with age of CTS or TF diagnosis after adjusting for accelerating covariates.

**Results:**

In total, 259 patients met the inclusion criteria. Our analysis revealed that patients with a BMI above 30 were diagnosed with CTS or TF an average of 6.4 years earlier than those with a normal BMI, at 67.5 and 61.1 years, respectively (*P* = .0053). Furthermore, covariate analysis showed that a BMI above 30 was associated with the onset of CTS or TF 6.2 years sooner than patients with normal BMI (*P* < .05). Current smokers developed CTS or TF 12.5 years sooner than never smokers (*P* < .05). Patients with type 1 diabetes developed CTS or TF, on average, 18.8 years sooner than nondiabetic patients (*P* < .05). Patients with hypertension developed CTS or TF on average 6.0 years later than those without hypertension (*P* < 0.05).

**Conclusions:**

Patients with a high BMI above 30 were diagnosed with CTS or TF at a younger age than normal BMI after covariate adjustment. Obesity is associated with accelerated development of CTS and TF after adjusting for comorbidities such as smoking and type 1 diabetes.

**Clinical relevance:**

This study sheds light on the earlier development of CTS and trigger finger in patients with a BMI greater than 30, contributing to a relatively underexplored area of upper-extremity musculoskeletal pathology research and enabling clinicians with more thorough data for preventative talks with patients.

Carpal tunnel syndrome (CTS) and trigger finger (TF) are highly prevalent upper-extremity disorders impacting millions nationwide. Determining the exact prevalence of CTS has been challenging due to its variable and transient presentation. Nonetheless, a review of the 1998 national health interview survey saw an estimated 2.65 million people report a diagnosis of CTS.^1^ Comparatively, TF, particularly trigger thumb, was estimated to affect over 200,000 individuals in the United States in 2021.^2^ These figures highlight the considerable impact of CTS and TF on public health and their frequent occurrence in orthopedic-hand clinic referrals.^3^ Understanding the pathophysiology, risk factors, and treatment options for CTS and TF is crucial for providing comprehensive patient care.

Carpal tunnel syndrome is characterized as a median nerve entrapment neuropathy at the wrist, resulting in pain, numbness, and paresthesia in the thumb, index finger, middle finger, and/or radial half of the ring finger.^4^ The causes of CTS are categorized as idiopathic—linked to known risk factors such as genetic predisposition, history of repetitive high-force wrist movements, metabolic syndrome, pregnancy, and autoimmune disorders—or secondary, related to anatomic abnormalities of the tenosynovium, nerve, and walls. Investigations of idiopathic risk factors have demonstrated a clear statistical association between CTS and factors such as female sex, age, and obesity.^5^ Additionally, metabolic syndrome, which constitutes a spectrum of obesity, cholesterol abnormalities, elevated blood pressure, and fasting glycemia, has been strongly associated with both CTS and TF.^6^ Metabolic syndrome when compared with diabetes, hypertension, and dyslipidemia had the greatest association with development of TF than the compared conditions alone.^7^ Notably, metabolic syndrome is associated not only with the development of CTS but also with its severity.^8^

Previous research has identified a connection between increased body mass index (BMI) and CTS and TF, but the relationship is often defined as a mediator of confounding variables.^9^, ^10^, ^11^, ^12^, ^13^, ^14^, ^15^ There is a paucity of data analyzing obesity and the temporal relationship of CTS or TF/thumb development. Our goal is to further the understanding of obesity and the development of CTS and TF.

## Materials and Methods

Institutional Review Board approval was obtained to conduct a retrospective chart review for adult patients diagnosed with CTS and/or TF between January 2016 and December 2020 from a single surgeon patient cohort at a large outpatient academic practice. Charts were selected based on International Classification of Diseases, 10th Revision (ICD-10) diagnosis codes of carpal tunnel: G56.00 (unspecific), G56.01 (right), G56.02 (left), G56.03 (bilateral), and TF: M65.30-M65.350. The primary outcome measure was age at first diagnosis of CTS or TF per records collected from a single surgeon’s practice database. Covariates include BMI, a minimum of 3 years, laterality of CTS or TF, number of trigger finger injections, number of carpal tunnel release surgeries, number of trigger finger release surgeries, smoking status, diabetes history, and hemoglobin A1c levels.

### Statistical methods

Summary statistics were produced for demographics and clinical parameters by BMI grouping. A normal (15–24.9 BMI) and high BMI group (30.0+ BMI) were generated, and each parameter was compared to determine multivariate regression model adjustments. Next, we generated BMI history in a categorical fashion of 3 years, 4–9 years, and 10+ years. We used a simple regression model to determine the difference in age at diagnosis between the normal and high BMI groups. We included adjustments for BMI history, smoking status, diabetic status, hypertension, and prediabetes in a multivariate regression model. Data cleaning was performed to assess patients with concomitant trigger finger/thumb and CTS. Patients with concomitant disease were filtered in RedCap, and the earliest respective diagnosis was included in the data analysis.

## Results

**Figure 1:**
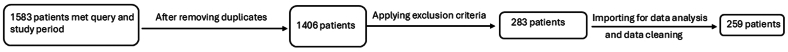
ICD-10 retreival and patient chart selection process.

We identified 259 patients with an average age of 62.2 years that met the inclusion criteria and the inquiry time frame for this study (Fig. 1). Our cohort was predominantly women (81.4%) with an average BMI of 30.1 (Table 1). There were 91 carpal tunnel and 168 TF diagnoses.

**Table 1:** Demographics and Clinical Parameters by BMI Group

Demographics and Clinical Parameters	Normal BMI	High BMI (30+)	Combined	*P* < .05
Age (y)	67.5 (13.9)	61.1 (12.6)	62.2 (13.0)	**.0053**
Sex (% f)	84.6%	79.3%	81.4%	.308
BMI	23.1	31.8	30.1	**<.001**
CTS (n)	16	75	91	.293
TF (n)	36	132	168	.247
Type 2 diabetes (n)	6 (14.0%)	57 (33.0%)	63 (29.2%)	.107
EMG utilization for CTS (%)	4 (33.3%)	45 (60.0%)	53.8%	.125
CTS right side	5 (16.1%)	26 (83.9%)	31 (100%)	.872
CTS left side	2 (13.3%)	13 (86.7%)	15 (100%)	
CTS bilateral	9 (19.6%)	37 (80.4%)	46 (100%)	
CTS injections	0.39	0.52	0.49	.6286
Trigger injections	2.71	2.72	2.72	.9848

Bold indicates *P* value <.05.

We used a bivariate linear regression to evaluate age at diagnosis for normal BMI versus high BMI. We found the normal BMI group developed CTS or TF at 6.4 years later than the high BMI group (95% confidence interval [CI]: 1.9, 10.9, *P* .005; Table 2).

**Table 2:** Linear Regression With Age at Diagnosis as the Outcome and Normal Versus High BMI

Age at Diagnosis	Coefficient	t	*P* > t	[95% CI]	
High BMI	−6.4	−2.82	.005	−10.9	−1.9
Normal BMI, mean age	67.4	32.48	.000	63.3	71.6

A multivariate regression model was used to evaluate the impact of significantly different covariates and factors that predispose individuals to develop CTS or TF; full data set is included in Table 3. We found that, in the presence of smoking status, current smokers developed CTS or TF 12.5 years (95% CI: 4.2, 20.8) earlier than never smokers. Former smokers and occasional smokers did not develop CTS or TF significantly sooner or later than never smokers, although the size of these subgroups was small. Patients with type 1 diabetes developed CTS or TF 18.8 years (95% CI: 10.1, 18.8) earlier than nondiabetic patients. Prediabetic and type 2 diabetes patients did not develop CTS or TF earlier or later than nondiabetic patients. Patients with hypertension developed CTS or TF 6.0 years (2.4, 9.5) later than patients without hypertension. We incorporated BMI history into the regression model but found that it did not significantly impact the development of CTS or TF. Patients with high BMI developed CTS or TF 6.2 years (95% CI: 1.8, 10.6) earlier than normal BMI patients after adjusting for the aforementioned covariates.

**Table 3:** Covariate Analysis of Smoking Status, Type 1 Diabetes, Type 2 Diabetes, and Hypertension

Covariates	Coefficient	[95% CI]		*P* < .05
High BMI	−6.2	−10.6	−1.8	**.006**
Smoking status				
Never smoker	Ref.	-	-	-
Current smoker	−12.5	−20.8	−4.2	**.003**
Former smoker	3.4	−0.08	6.9	.055
Occasional smoker	8.6	−5.3	22.4	.225
Diabetic status				
Not diabetic	Ref.	-	-	-
Type 1	−18.8	−27.4	−10.1	**<.001**
Type 2	−0.5	−4.4	3.4	.799
Prediabetes	3.4	−8.8	15.7	.586
Hypertension	6.0	2.4	9.5	**.001**
Intercept (mean)	64.8	60.5	69.1	**<.001**

Bold indicates *P* value <.05.

## Discussion

The most important finding of this study was that patients with a body mass index over 30 developed CTS and TF significantly earlier compared with patients with a BMI less than 25. Notably, factors like a history of type 2 diabetes or a longer history of elevated BMI did not show a significant relationship with the development of these conditions. Although previous research has established a connection between obesity and lower-extremity musculoskeletal pathologies, our study investigated these phenomena for non–weight-bearing upper-extremity pathologies and hypothesized a similar correlation for CTS and TF.

Our analysis indicated that patients with a BMI above 30 developed CTS or TF 6.4 years earlier than patients with a normal BMI. These findings algin with a 2002 case control study by Becker et al,^5^ which also observed a higher frequency of CTS in individuals with a BMI greater than 30. Additionally, this study saw that the proportion of patients in the case group was between ages 41 and 60 compared with the control group, with ages varying between 18 and 90 years, highlighting the insidious impact BMI has on the development of CTS. A 2012 electrophysiologic study by Onder et al^6^ analyzed the severity of CTS between patients with metabolic syndrome and those without. The metabolic syndrome group had a higher percentage of patients with moderate and severe neurophysiological defined CTS compared with the group without metabolic syndrome, emphasizing the impact of obesity on the severity of the condition.^6^

The association between high BMI and the development of CTS and TF has been consistently supported by various studies. However, the effect of weight loss on symptom improvement or changes in median nerve neurophysiology remains under-researched. For instance, a study by Kurt et al^16^ found no significant changes in nerve conduction studies after weight loss in patients with CTS, despite a significant reduction in their BMI. This highlights the necessity for further research into the inflammatory environment associated with high BMI, the role of specific inflammatory mediators, and their potential irreversible effects on CTS and TF progression.

Our study also considered covariates such as diabetes, smoking status, and hypertension. We observed significant differences in the development time of CTS and TF related to these factors, with onset times of −18.8, −12.5, and +6.5 years, respectively. A 2016 meta-analysis suggested that both type 1 and type 2 diabetes are risk factors for the development of CTS.^17^ However, more recent studies, including ours, question this association, especially regarding type 2 diabetes as an independent risk factor. Low et al^18^ investigated the association between diabetes mellitus and CTS using over 322,092 patient records, and after adjusting for confounding variables, they found no statistically significant correlation. Conversely, Wilburg et al,^19^ using a United Kingdom-based cohort of over 400,000 patients, had an odds ratio of 2.31 with a 95% CI of 2.17–2.46, suggesting a strong positive connection between diabetes and CTS development. A recently published 2024 meta-analysis, which included 42 studies across 35 years and 3 million patients, reported a modified odds ratio of 1.68 (95% CI: 1.45–1.94), suggested a positive correlation between the two states and found no difference when stratifying by type 1 versus type 2 diabetes.^20^

Retrospective cohort studies can only establish correlation; however, the reproducibility of these results across other studies highlights the strong correlation between inflammatory states and CTS and TF. However, the pathophysiologic mechanism remains elusive, with varying theories being currently investigated. Previous research has elucidated obesity as a progenitor to a chronic grade inflammatory state that induces an increase in white adipose proinflammatory cytokines, TNF-alpha, and IL06.^21^ A 2020 study saw that IL-6 overproduction by tenosynovial fibroblasts may have accounted for the association of CTS and TF in patients.^22^ In 2014, Mojadidi et al^23^ biopsied over 40 posterior interosseous nerves in diabetic and nondiabetic patients undergoing carpal tunnel release and saw a prolonged distal motor latency with a decreased axon diameter, axon area, and myelinated fiber density in diabetic patients. Furthermore, the expression of vascular endothelial growth factor A and its receptors was significantly increased, especially in type 1 diabetes, compared with nondiabetic patients, suggesting a space-filling vascular growth, leading to increased nerve compression in the carpal tunnel.^23^ A different theory postulates that the increased production of advanced glycation end products in diabetes leads to cross-linking of macromolecules in the extracellular matrix, resulting in increased area of extracellular matrix and vascular stiffness.^24^

It is important to note that our study relied on ICD-10 codes for data collection, which can be influenced by factors like accurate documentation and billing. Dependence of electronic medical records also means that the quality and accuracy of the data are contingent upon correctly available patient records. Accuracy of data entry is subject to human error by these authors, which can impact study results. Carpal tunnel syndrome remains primarily a clinical diagnosis, and thus, a portion of patients may have been inappropriately diagnosed prior to referral and included in this study. Additionally, the analysis of the impact of a long history of elevated BMI on the development of CTS and TF was limited by a smaller sample size than the normal BMI group, potentially because of lower health care utilization. Furthermore, the inability to collect data on patients’ occupations, which may contribute to the development of CTS, especially in professions involving repetitive wrist motions, is a notable limitation. This study sheds light on the earlier development of CTS and TF in patients with a BMI greater than 30, contributing to a relatively underexplored area of upper-extremity musculoskeletal pathology research. Our findings provide valuable insights for health care providers, enabling more informed and evidence-based discussions about weight management and the prevention of musculoskeletal disorders. Continued research in this field is essential to deepen our understanding of the pathophysiological connections between obesity, inflammation, and synovial diseases.

## Conflicts of Interest

No benefits in any form have been received or will be received related directly to this article.

## References

[1] Lawrence R.C., Felson D.T., Helmick C.G. (2008). Estimates of the prevalence of arthritis and other rheumatic conditions in the United States. Part II. Arthritis Rheum.

[2] Pencle F., Harberger S., Tiwari V., Molnar J.A. (2025).

[3] Wildin C., Dias J.J., Heras-Palou C., Bradley M.J., Burke F.D. (2006). Trends in elective hand surgery referrals from primary care. Ann R Coll Surg Engl.

[4] Sevy J.O., Sina R.E., Varacallo M.A. (2025).

[5] Becker J., Nora D.B., Gomes I. (2002). An evaluation of gender, obesity, age and diabetes mellitus as risk factors for carpal tunnel syndrome. Clin Neurophysiol.

[6] Onder B., Yalçın E., Selçuk B., Kurtaran A., Akyüz M. (2013). Carpal tunnel syndrome and metabolic syndrome co-occurrence. Rheumatol Int.

[7] N J.H.S., L A.H.A.F., R G.V.G., da Silveira D.C.E.C., B P.N., Almeida S.F. (2021). Epidemiology of trigger finger: metabolic syndrome as a new perspective of associated disease. Hand (N Y).

[8] Gül Yurdakul F., Bodur H., Öztop Çakmak Ö. (2015). On the severity of carpal tunnel syndrome: diabetes or metabolic syndrome. J Clin Neurol.

[9] Mansoor S., Siddiqui M., Mateen F. (2017). Prevalence of obesity in carpal tunnel syndrome patients: a cross-sectional survey. Cureus.

[10] Chen J.Q., Wang D., Liu B. (2023). Body mass index and carpal tunnel syndrome: a case-control study. Medicine (Baltimore).

[11] Adebayo P.B., Mwakabatika R.E., Mazoko M.C., Taiwo F.T., Ali A.J., Zehri A.A. (2020). Relationship between obesity and severity of carpal tunnel syndrome in tanzania. Metab Syndr Relat Disord.

[12] Balci K., Utku U. (2007). Carpal tunnel syndrome and metabolic syndrome. Acta Neurol Scand.

[13] Vernick R.C., Beckwitt C.H., Fowler J.R. (2024). Subjective and objective differences in patients with unilateral and bilateral carpal tunnel syndrome and the role of obesity in syndrome severity. Plast Reconstr Surg.

[14] Werner R.A., Albers J.W., Franzblau A., Armstrong T.J. (1994). The relationship between body mass index and the diagnosis of carpal tunnel syndrome. Muscle Nerve.

[15] Seror P., Seror R. (2013). Prevalence of obesity and obesity as a risk factor in patients with severe median nerve lesion at the wrist. Joint Bone Spine.

[16] Kurt S., Kisacik B., Kaplan Y., Yildirim B., Etikan I., Karaer H. (2008). Obesity and carpal tunnel syndrome: is there a causal relationship?. Eur Neurol.

[17] Pourmemari M.H., Shiri R. (2016). Diabetes as a risk factor for carpal tunnel syndrome: a systematic review and meta-analysis. Diabet Med.

[18] Low J., Kong A., Castro G., Rodriguez de la Vega P., Lozano J., Varella M. (2021). Association between diabetes mellitus and carpal tunnel syndrome: results from the united states national ambulatory medical care survey. Cureus.

[19] Wiberg A., Smillie R.W., Dupré S., Schmid A.B., Bennett D.L., Furniss D. (2022). Replication of epidemiological associations of carpal tunnel syndrome in a UK population-based cohort of over 400,000 people. J Plast Reconstr Aesthet Surg.

[20] Sanjari E., Raeisi Shahraki H., G Khachatryan L., Mohammadian-Hafshejani A. (2024). Investigating the association between diabetes and carpal tunnel syndrome: a systematic review and meta-analysis approach. PLoS One.

[21] Kern L., Mittenbühler M.J., Vesting A.J., Ostermann A.L., Wunderlich C.M., Wunderlich F.T. (2018). Obesity-induced TNFα and IL-6 signaling: the missing link between obesity and inflammation-driven liver and colorectal cancers. Cancers (Basel).

[22] Kawamoto H., Iwatsuki K., Kurimoto S. (2020). Interleukin-6 secretion by fibroblasts in carpal tunnel syndrome patients is associated with trigger finger and inhibited by tranilast. Muscle Nerve.

[23] Mojaddidi M.A., Ahmed M.S., Ali R. (2014). Molecular and pathological studies in the posterior interosseous nerve of diabetic and non-diabetic patients with carpal tunnel syndrome. Diabetologia.

[24] Goldin A., Beckman J.A., Schmidt A.M., Creager M.A. (2006). Advanced glycation end products: sparking the development of diabetic vascular injury. Circulation.

